# Complete Genome Sequence of the Bacterial Component of Mysorin Biopreparation

**DOI:** 10.1128/MRA.01287-20

**Published:** 2021-03-18

**Authors:** Alexey M. Afonin, Emma S. Gribchenko, Gulnar A. Akhtemova, Yuri V. Laktionov, Andrej P. Kozhemyakov, Vladimir A. Zhukov

**Affiliations:** a All-Russia Research Institute for Agricultural Microbiology (ARRIAM), Saint Petersburg, Russian Federation; University of Arizona

## Abstract

Plants can form various beneficial associations with soil microorganisms, such as associations with plant growth-promoting bacteria (PGPB). In this work, we report the full-genome sequence of the component of Mysorin biopreparation, identified as Microbacterium hominis, consisting of a single 3.5-Mbp circular chromosome.

## Abstract (precis)

Plants can form various beneficial associations with soil microorganisms, such as associations with plant-growth-promoting bacteria (PGPB). In this work, we report the full genome sequence of the component of Mysorin biopreparation, identified as Microbacterium hominis, consisting of a single 3.5-Mbp circular chromosome.

## ANNOUNCEMENT

The strain was originally isolated form the rhizosphere from an *Arrhenatherum* sp. plant in 1979 and was shown to possess plant growth-promoting (PGP) qualities (1). It is a component of the “Mysorin” biopreparation used to promote root growth in various crops (https://ekosgroup.ru/catalog/gruppy-preparatov/biopreparaty-dlya-pitaniya-rostostimulyatsii-i-rostoregulyatsi/mizorin/). The strain was deposited in the Russian Collection of Agricultural Microorganisms (RCAM) in the All-Russia Research Institute for Agricultural Microbiology under the accession number RCAM 01094 and the name Arthrobacter mysorens 7. The genome of the strain was not previously investigated.

For the DNA isolation, a sample of the bacteria used in the production line of Mysorin preparation was cultivated in 50 ml of liquid tryptone-yeast (TY) medium (28°C, 180 rpm) (2). Culture was harvested after 24 h of incubation. DNA for library preparation for both the short-read and long-read sequencing was isolated using the phenol-chloroform method according to reference 3.

Long-read sequencing was performed using a MinION sequencer at the All-Russia Research Institute for Agricultural Microbiology (ARRIAM). The SQK-LSK109 kit using the LFB buffer from the kit for short-read elimination and barcode number 3 from the EXP-NBD104 barcoding kit were used to prepare the library according to the manufacturer’s instructions, omitting the DNA-shearing step. The reads were base called and demultiplexed using the guppy_basecaller v. 3.3.0. The resulting read *N*_50_ value was 20,027 bp, with a total read length of 1.15 gigabases.

Short-read whole-genome sequencing was carried out by Macrogen, Inc. (Seoul, South Korea), on an Illumina platform using the TruSeq DNA PCR-free kit; in total, 12.1 million 2 × 150-bp sequence reads were generated. The reads were quality trimmed, and adapter sequences and possible contaminants were removed as previously described (4).

Flye v. 2.6-release (5) was used to assemble the nanopore reads. The resulting assemblies were corrected using Racon v. 1.3.2 with the following modifiers: -m 8 -x -6 -g -8 -w 500 (6); they were then polished using Medaka v. 0.10.0 and 5 iterative rounds of polishing with short reads using Pilon v. 1.22 (7).

The genome consists of a single chromosome of 3,475,666 bp (GC content, 71%; long-read coverage, 327×; short-read coverage, 509×). PGAP v. 4.13 (8) was used to annotate the assembled transcripts; it contains 3,280 protein coding genes, 2 rRNA operons, and 47 tRNA genes.

To determine the taxonomy, 16S rRNA and DNA gyrase B genes were searched with BLAST against the nonredundant (nr) NCBI database, with Microbacterium hominis strain SJTG1 being the closest strain. The average nucleotide identity (ANI) comparison to the genomes of *M. hominis* present in the NCBI database as calculated with fastANI (https://github.com/ParBLiSS/FastANI) showed the most similar strains to be the type strain NBRC_15708 (98.8738%) and strain SJTG1 (98. 8561%), in contradiction to the previous descriptions of the strain.

The genome was investigated for PGP-associated genes. The BAGEL4 Web server (9) found no areas of interest; a blastp search (10) against PHI-base (11) and both the C and KS parts of the NaPDoS database (12) yielded no results. The antiSMASH v. 5 pipeline (13) showed three secondary metabolite regions, NPRS-like, terpene, and beta-lactone. Although all three regions can potentially be responsible for plant growth-promoting activity, the mechanism is not immediately apparent. Further investigation of this strain in plant associations can possibly lead to the discovery of novel methods of plant growth promotion in soil.

### Data availability.

The genome sequence of the strain was deposited in GenBank under the accession number CP061344.1. The raw sequencing data are registered in the NCBI SRA database under the accession numbers SRR12502383 (short-read data) and SRR12502384 (long-read data). This announcement describes the first version of the assembly.
